# Disappearance of treatment-resistant depression after damage to the orbitofrontal cortex and subgenual cingulate area: a case study

**DOI:** 10.1186/s12883-016-0717-x

**Published:** 2016-10-19

**Authors:** Michitaka Funayama, Motoichiro Kato, Masaru Mimura

**Affiliations:** 1 Department of Neuropsychiatry, Ashikaga Red Cross Hospital, Ashikaga-City, 326-0843 Japan; 2 Department of Neuropsychiatry, Keio University School of Medicine, 160-8582 Tokyo, Japan

**Keywords:** Treatment-resistant depression, Regret, Subarachnoid hemorrhage, Orbitofrontal cortex, Subgenual cingulate area

## Abstract

**Background:**

Although post-stroke depression is a well-characterized disorder, there is less understanding of how pre-existence of depression is affected by a stroke.

**Case presentation:**

We describe a patient with treatment-resistant major depression, which had been ongoing for 14 years but disappeared shortly after onset of a subarachnoid hemorrhage. Her cognitive function and functional status were mostly unaffected by the stroke. However, she no longer excessively regretted past events. Lesions were found in the orbitofrontal cortex, which is involved in feeling regret, and in the adjacent subgenual cingulate area, which is metabolically hyperactive in treatment-resistant depression and is the target for deep-brain stimulation for relief of treatment-resistant depression. The lesions from the stroke may have caused the disappearance of the patient’s treatment-resistant depression by alleviating excessive regret and decreasing the elevated activity in these areas.

**Conclusions:**

This patient’s clinical course may shed light on the neuropsychological and neurophysiological mechanisms of major depression of the melancholic subtype.

## Background

Although post-stroke depression is a well-known disorder, how a pre-existing depression is affected by stroke has not been well studied. To the best of our knowledge, only Mimura et al. [1] have documented the post-stroke character of a patient who had major depression before the stroke event. This patient showed acute mania following a right-side infarction in the area of the middle cerebral artery. Studies on mood changes after brain damage can help elucidate the brain mechanisms that regulate mood states. Here, we describe a female patient with treatment-resistant major depression that had lasted for 14 years but improved dramatically shortly after a subarachnoid hemorrhage with the rupture of an anterior-communicating artery aneurysm. We discuss potential mechanisms underlying her mood change.

## Case presentation

The patient and her family members granted informed consent in accordance with the Declaration of Helsinki. Ethical aspects of this study were reviewed and approved by the Ashikaga Red Cross Hospital Human Research Ethics Committee. The patient was a 69-year-old housewife with 14 years of education and no remarkable medical history. Her parents ran a family-owned precision instruments plant. She married an employee of the company, who later succeeded her father as president. She was a serious-minded person who liked order and was dedicated to her husband and the management of the family firm. She would sacrifice her well-being for others and had a strong sense of responsibility. She tended to think and plan seriously before acting. She first presented with depression at age 55, shortly after her father-in-law died. Although she had taken care of him for the preceding five years, she regretted not having been able to positively help him during his illness. She visited the Ashikaga Red Cross hospital at that time for help with her depressive state, and we followed her thereafter. The patient’s symptoms included a severely depressed mood, anhedonia, poor concentration, low energy, psychomotor retardation, irritability, agitation, insomnia, feelings of excessive guilt, and suicidal ideation. She was diagnosed with major depression of the melancholic type [2] and was prescribed the tricyclic antidepressant trimipramine maleate, which was gradually increased to 150 mg daily over a period of 3 months. Although her depression partially remitted during the course of treatment, it deteriorated again at age 57 when her mother-in-law was diagnosed with dementia and admitted to a nursing home. The patient again had excessive feelings of regret about not having effectively helped her mother-in-law. She was prescribed the tricyclic antidepressant, amoxapine (150 mg daily), but her depression did not improve. Indeed, it deteriorated further between age 62 and 68 during which time she lost her three siblings. At age 68, her scores for depression on the Hamilton [3] and MADRS-J [4] scales were 28 and 36, respectively, which characterized her as very severely depressed (Table 1). Although the serotonin and norepinephrine reuptake inhibitor milnacipran (100 mg daily) was used with the amoxapine, her symptoms did not change. She often stayed indoors, and on her last visit to our hospital two weeks prior to the subarachnoid hemorrhage at the age of 69, her scores for depression on the Hamilton and MADRS-J scales were 29 and 38, respectively (Table 1).

**Table 1:** The patient’s scores for depression on the Hamilton [3] and MADRS-J [4] scales

	68 years	69 years	69 years (2 months post-onset)	70 years (1 year post-onset)
Hamilton [3]	28	29	6	4
MADRAS-J [4]	36	38	5	5

Age when tested is given in the first line of the table

**Fig. 1:**
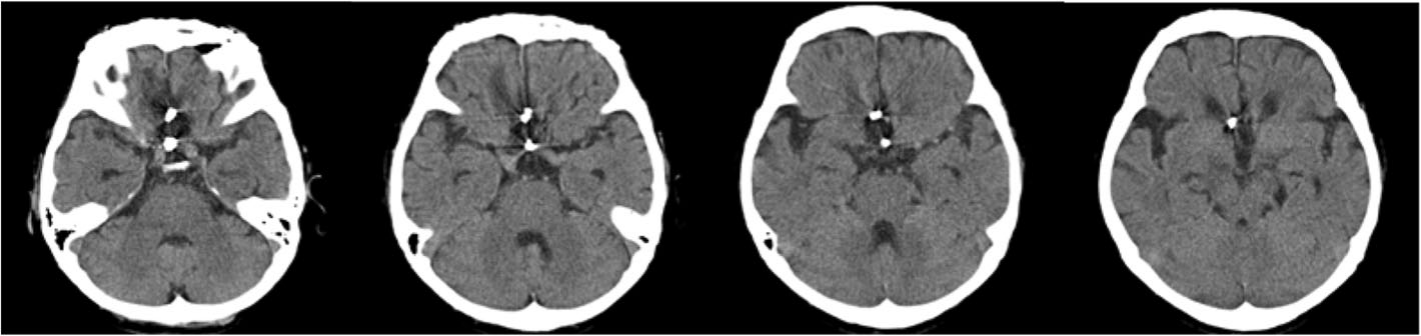
Computed tomography imaging of the patient’s head, showing a low-density area in the bilateral orbitofrontal cortex subgenual cingulate area and surgical clippings for the rupture of the anterior-communicating artery aneurysm and to prevent rupture of the right anterior cerebral artery aneurysm

**Fig. 2:**
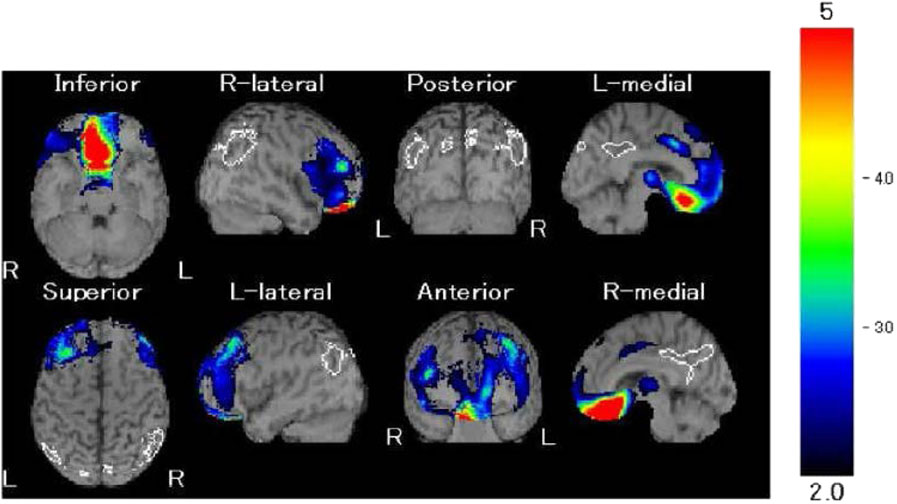
Hypoperfusion was demonstrated in the orbitofrontal cortex, basal forebrain, and subgenual cingulate areas using IMP SPECT of the Easy Z-Score Imaging System, which is a voxel-by-voxel Z-score analysis after voxel normalization to the global mean values. Z-score = ([control population mean] – [individual value]/[control population standard deviation]), as displayed on the anatomically standardized MRI template. The colored areas demonstrate a significant decrease in regional cerebral blood flow. For example, red corresponds to a Z-score of −4.5 or less negative, i.e., most severely decreased, and blue corresponds to a Z-score between −3 and −2.5, i.e., less severely decreased

The subarachnoid hemorrhage (Hunt and Hess grade 3 [5]), which involved the rupture of an anterior-communicating artery aneurysm, required surgical clipping. The computed tomography of her head (Fig. 1) demonstrated a low-density area in the orbitofrontal cortex and subgenual cinguate area, which was apparently caused by an intraparenchymal hemorrhage. The imaging also shows a surgical clipping for the ruptured aneurysm and a second clipping to prevent further rupture of the right anterior cerebral artery aneurysm. We evaluated the patient’s regional cerebral blood flow with IPM single-photon emission tomography using the Easy Z-Score Imaging System [6], which is a voxel-by-voxel Z-score analysis after voxel normalization to global mean values. The Z-score = ([control population mean] – [individual value]/[control population standard deviation]), is displayed on the anatomically standardized MRI template (Fig. 2). The imaging demonstrates dense hypoperfusion in the orbitofrontal cortex, basal forebrain, and subgenual cingulate area.

No complications common to a subarachnoid hemorrhage (i.e., hydrocephalus and/or vasospasm) were observed, and the patient recovered sufficiently to be discharged from the hospital after 2 months. She had no palsy or sensory disturbance. Antidepressant treatments were stopped immediately after her admission and were never resumed. For several months after the stroke, she was forgetful and showed mild spontaneous confabulation. Eventually, her memory deficit and confabulation improved, and she began living independently 6 months post-onset. She and her family members said that she had no cognitive dysfunction and was no longer forgetful.

Her cognitive function was evaluated 6 months after the hemorrhage using a wide range of neuropsychological measures. Her general cognitive function was assessed using the Japanese version of the Mini Mental State Examination [7], on which she had a perfect score of 30/30. The Japanese version of the Wechsler Adult Intelligence Scale [8] was used to evaluate her verbal and performance intelligence quotients, and her respective scores were 103 and 97, both within the normal range of 70 to 130 and close to the average of 100. For executive function, the Japanese version of the Behavioral Assessment of the Dysexecutive Syndrome [9] was used, and her score of 109 was within the normal range of 70 to 130 and slightly above the average of 100. Her episodic memory and attentional function were assessed using the Japanese version of the Wechsler Memory Scale-Revised [10], for which she scored 84 for verbal memory, 86 for visual memory, 80 for delayed recall, and 109 for attention/concentration, all within the normal range of 70 to 130. However, compared with both the Wechsler Adult Intelligence and the Behavioral Assessment of the Dysexecutive Syndrome scales, her episodic memory scores were relatively small, suggesting that she might have a very mild memory deficit. Finally, the Japanese version of the Frenchay Activities Index [11] was used to evaluate her functional status when using instruments of daily living activities, e.g., housework. Her score of 29 was within the normal range of 27.5 ± 8.6 for females age 70–79 years. In summary, although no pre-onset scores are available for comparison with the scores 6 months after the hemorrhage, her cognitive and functional status was considered to be mostly unaffected.

Her depression disappeared completely shortly after the stroke. Her scores on the Hamilton scale and MADRS-J at 2 months post-hemorrhage decreased (i.e., improved) to 6 and 5, respectively, both of which are within the normal range (Table 1). She performed housework efficiently, often went out with her friends, and had no manic episodes. She also did not exhibit apathy, disinhibition, or impulsiveness, which are frequently observed after damage to the orbitofrontal cortex. Her serious-minded and self-sacrificing behaviors and strong sense of responsibility lessened, but she did not become irresponsible. She also no longer excessively regretted past events. Her family members reported that before the stroke she had spent much time regretting past events and that her behavior and life changed in positive ways after the stroke. When asked if the experience of a serious physical illness and a narrow escape from death had changed her way of thinking, she replied that she did not agree with that statement and that she did not know why her way of thinking had changed. She said that she used to live an excessively organized and orderly life before the stroke, but somehow no longer took things too seriously. At age 70, 1 year after the stroke, her scores on the Hamilton scale and MADRS-J were four and five, respectively, within the normal range (Table 1). She had no recurrence of depression for 10 years after the stroke.

## Conclusions

We describe herein a patient with treatment-resistant major depression that lasted for 14 years and that improved dramatically shortly after a subarachnoid hemorrhage, which disrupted the orbitofrontal cortex and subgenual cingulate area. Following the stroke, she no longer experienced excessive regret for past events. Possibly, the change in her behavior reflects a normal psychological response to a severe physical illness. However, elderly people are generally unable to change their behavior, and this patient did not agree with this suggestion. In our opinion, her 10-year recovery from a depressive state that had lasted for 14 years prior to the stroke cannot be explained as merely a psychological response.

Mood change after brain damage has been reported previously. Gainotti [12] found that patients with left-sided lesions were more likely to demonstrate emotional tendencies for “catastrophic reactions” (anxiety, tears, and/or swearing), and those with right-sided lesions were more likely to show “indifference reactions” (indifference, minimization, and/or anosognosia). Right-hemisphere lesions are also sometimes associated with undue cheerfulness [13] and euphoric mood change [14]. These past studies suggest that right-hemisphere lesions might result in a positive mood. However, this is not the case for this patient, whose lesions were not limited to the right hemisphere. Rather, the patient’s lesions involved the bilateral orbitofrontal cortex and the subgenual cingulate region. According to Camille and colleagues [15], the orbitofrontal cortex is involved in the experience of regret. Before her subarachnoid hemorrhage, the patient’s depression was considered to be the melancholic subtype [2], with a tendency for an extreme sense of order and responsibility, and excessive regret about the past. Thus, possibly, the patient’s loss of excessive regret was caused by damage to the orbitofrontal cortex.

In addition to the aforementioned neuropsychological mechanism, another potential mechanism for the patient’s clinical course involves brain metabolism. According to Mayberg and colleagues [16], the subgenual cingulate area may be critically involved in modulating negative mood states and is metabolically hyperactive in patients with treatment-resistant depression. In support of this hypothesis, a decrease in subgenual cingulate activity has been reported in patients who responded to antidepressant treatments or had ablative surgery [17, 18]. Furthermore, her group assessed whether chronic deep brain stimulation could decrease subgenual cingulate activity and produce a clinical benefit in six patients with refractory depression [19]. Using chronic stimulation of white matter tracts adjacent to the subgenual cingulate area, a striking and sustained remission of depression was obtained for four of the six patients who also showed decreased regional cerebral blood flow in the subgenual cingulate area and orbitofrontal cortex. Recently, Hilimire et al. [20] reported that subgenual cingulate deep brain stimulation reduced negative self-bias in patients with treatment-resistant depression and the change in self-bias may contribute to changes in mood. Given the role of the subgenual cingulate area in mood regulation and the fact that it was also disrupted in our patient, this area in our patient perhaps was metabolically hyperactive prior to the hemorrhage, and damage to the region and its adjacent orbitofrontal cortex reduced this elevated activity leading to the complete disappearance of her depression.

The aforementioned neuropsychological and metabolic mechanisms are not incompatible. Rather, we consider that damage to the orbitofrontal cortex and the adjacent subgenual cingulate area may have worked together to produce the disappearance of the treatment-resistant depression by alleviating her excessive regret and metabolic hyperactivity. Although the neural basis for depression is still incompletely understood, the orbitofrontal cortex and anterior cingulate are among the areas that are most consistently implicated in depression, as are the prefrontal cortex, amygdala, medial temporal lobe, striatum, thalamus, and brain stem [21, 22]. The details of our case study cannot explain the neural basis of depression, but it suggests intriguing neuropsychological and neurophysiological mechanisms underlying some cases with treatment-resistant depression of the melancholic subtype.

## References

[1] Mimura M, Nakagome K, Hirashima N, Ishiwata H, Kamijima K, Shinozuka A (2005). Left frontotemporal hyperperfusion in a patient with post-stroke mania. Psychiatry Res.

[2] Tellenbach H (1961). Melancholie. Zur Problemgeschichte, Typologie, Pathogenese und Klinik. Mit einem Geleitwort von V.E. von Gebsattel.

[3] Narita T, Kin N, Nakane M, Ozaki N (2003). Reliability and validity on structured interview guide for the Hamilton depression rating scale. Japanese Journal of Clinical Psychopharmacology.

[4] Inada T (2004). Clinical evaluation of depressive disorders by the MADRS Japanese version using the SIGMA.

[5] Hunt WE, Hess RM (1968). Surgical risk as related to time of intervention in the repair of intracranial aneurysms. J Neurosurg.

[6] Matsuda H, Mizumura S, Soma T, Takemura N (2004). Conversion of brain SPECT images between different collimators and reconstruction processes for analysis using statistical parametric mapping. Nucl Med Commun.

[7] Sugishita M, Hemmi I (2010). Validity and reliability of Mini Mental State Examination-Japanese (MMSE-J): a preliminary report. Japanese J Cogn Neurosci.

[8] Fujii K (2006). Wechsler Adult Intelligence Scale.

[9] Kashima H (2003). Behavioural Assessment of the Dysexecutive Syndrome.

[10] Sugishita M (2001). Wechsler Memory Scale-Revised.

[11] Hachisuka K, Chisaka H, Kawazu T, Saeki S, Negayama S (2001). Applied activities of daily living and its standard value determined according to Frenchay activities index scores for randomly sampled middle and advanced age people living at home. Japanese Journal of Rehabilitation Medicine.

[12] Gainotti G (1972). Emotional behavior and hemispheric side of the lesion. Cortex.

[13] Starkstein SE, Robinsion RG, Honig MA, Parkh RM, Joselyn J, Price TR (1989). Br J Psychiatry.

[14] Sackeim HA, Greenberg MS, Weiman AL, Gur RC, Hungerbuhler JP, Geschwind N (1982). Hemispheric asymmetry in the expression of positive and negative emotions. Neurologic evidence. Arch Neuro.

[15] Camille N, Coricelli G, Sallet J, Pradat-Diehl P, Jean-René D, Sirigu A (2004). The involvement of the orbitofrontal cortex in the experience of regret. Science.

[16] Mayberg HS, Liotti M, Brannan SK, McGinnis S, Mahurin RK, Jerabek PA (1999). Reciprocal limbic-cortical function and negative mood: converging PET findings in depression and normal sadness. Am J Psychiatry.

[17] Mayberg HS, Brannan SK, Mahurin RK, McGinnis S, Silva JA, Tekell JL (2000). Regional metabolic effects of fluoxetine in major depression: serial changes and relationship to clinical response. Biol Psychiatry.

[18] Dougherty DD, Weiss AP, Consgrove GR, Alpert NM, Cassem EH, Nierenberg A (2003). Cerebral metabolic correlates as potential predictors of response to cingulotomy for major depression. J Neurosurg.

[19] Mayberg HS, Brannan SK, Tekell JL, Silva JA, Mahurin RK, McGinnis S (2005). Deep brain stimulation for treatment-resistant depression. Neuron.

[20] Hilimire MR, Mayberg HS, Holtzheimer PE, Broadway JM, Parks NA, DeVylder JE, Corballis PM (2015). Effects of subcallosal cingulate deep brain stimulation on negative self-bias in patients with treatment-resistant depression. Brain Stimul.

[21] Pandya M, Altinay M, Malone DA, Anand A (2012). Where in the brain is depression?. Curr Psychiatry Rep.

[22] Kerestes R, Davey CG, Stephanou K, Whittle S, Harrison BJ (2014). Functional brain imaging studies of youth depression: a systematic review. Neuroimage: Clinical.

